# Cascade Aggregation Network for Accurate Polyp Segmentation

**DOI:** 10.1049/syb2.70036

**Published:** 2025-09-05

**Authors:** Yanru Jia, Yu Zeng, Huaping Guo

**Affiliations:** ^1^ School of Big Data and Artificial Intelligence Xinyang University Xinyang China; ^2^ School of Computer and Information Techonology Xinyang Normal University Xinyang China

**Keywords:** cascade aggregation, multiscale context aware, polyp segmentation

## Abstract

Accurate polyp segmentation is crucial for computer‐aided diagnosis and early detection of colorectal cancer. Whereas feature pyramid network (FPN) and its variants are widely used in polyp segmentation, inherent limitations existing in FPN include: (1) repeated upsampling degrades fine details, reducing small polyp segmentation accuracy and (2) naive feature fusion (e.g., summation) inadequately captures global context, limiting performance on complex structures. To address limitations, we propose a cascaded aggregation network (CANet) that systematically integrates multi‐level features for refined representation. CANet adopts PVT transformer as the backbone to extract robust multi‐level representations and introduces a cascade aggregation module (CAM) that enriches semantic features without sacrificing spatial details. CAM adopts a top‐down enhancement pathway, where high‐level features progressively guide the fusion of multiscale information, enhancing semantic representation while preserving spatial details. CANet further integrates a multiscale context‐aware module (MCAM) and a residual‐based fusion module (RFM). MCAM applies parallel convolutions with diverse kernel sizes and dilation rates to low‐level features, enabling fine‐grained multiscale extraction of local details and enhancing scene understanding. RFM fuses these local features with high‐level semantics from CAM, enabling effective cross‐level integration. Experiments show that CANet outperforms SOTA methods in in‐ and out‐of‐distribution tests.

## Graphical abstract

We propose CANet, a novel end‐to‐end segmentation network for polyp segmentation that integrates multiscale information fusion and spatial detail preservation. CANet consists of three key modules: CAM for semantic enhancement, MCAM for capturing fine‐grained details in complex backgrounds and RFM for integrating high‐ and low‐level features. Experimental results demonstrate that CANet exhibits strong learning ability and generalisation performance in both in‐distribution and out‐of‐distribution tests.

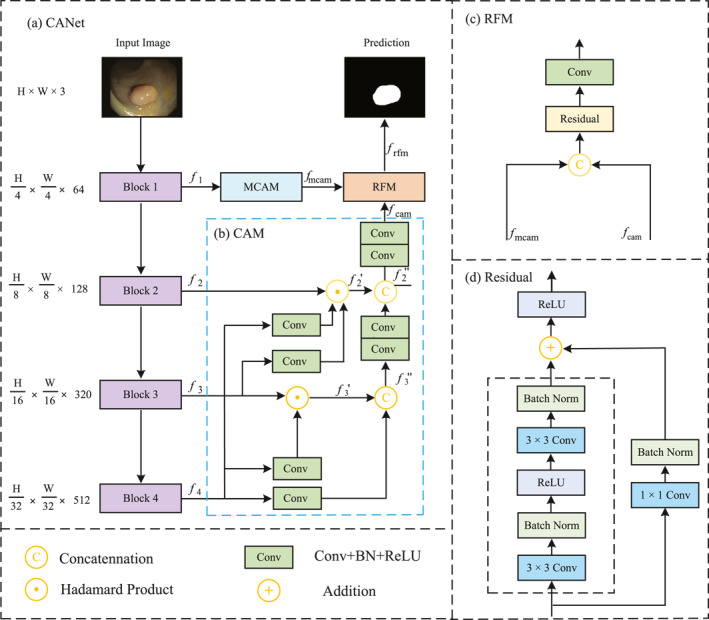

## 1 Introduction

Colorectal cancer (CRC) ranks among the most prevalent and deadly cancers globally, representing 10% of all cancer‐related deaths [1]. Notably, studies report polyp miss rates of 20%–30% during standard colonoscopies [2]. This high miss rate is likely related to the complex bowel structure and the difficulty in detecting small lesions [3]. Therefore, developing more accurate auxiliary detection technologies is crucial for improving diagnostic efficiency.

In recent years, with the rapid advancement of deep learning technologies, feature pyramid networks (FPNs) have emerged as a powerful approach in computer vision, particularly for medical image segmentation tasks [4]. By integrating feature maps from multiple layers, FPN effectively captures both global context and fine‐grained local details, significantly improving segmentation accuracy, especially in scenarios with complex backgrounds and variable target morphologies. As a typical FPN model, U‐Net [5] and its improved versions (such as Unet++ [6] and U‐Net 3+ [7]) have become foundational in modern segmentation frameworks, which leverage an encoder–decoder structure with skip connections to integrate high‐level semantic representations with low‐level spatial details, enabling accurate reconstruction of high‐resolution segmentation outputs.

Many methods based on U‐Net have been proposed for the polyp segmentation task [8, 9]. Jha et al. [10] proposed DoubleU‐Net, a cascaded architecture combining two U‐Nets, where the initial U‐Net output serves as the attention input for the subsequent U‐Net. DoubleU‐Net further integrates the squeezed excitation network and the spatial pyramid pooling with holes to enhance performance. Yu et al. [11] introduced an efficient dilated convolution structure that enriches contextual information, improving the accuracy of semantic segmentation models. Jha et al. [12] proposed TransRUPNet, a model built upon a pretrained pyramid vision transformer [13]. It consists of three encoder–decoder modules and integrates an upsampling module at the end to further improve polyp segmentation accuracy. The PolypSeg [14] method utilises a dual adaptive feature mechanism for multiscale modelling: deformable convolutions are used to dynamically adjust the receptive field for polyps of varying sizes, whereas channel attention mechanisms enable cross‐layer fusion, effectively suppressing noise in shallow features. MSRFNet [15] passes high‐ and low‐resolution features by integrating a cross‐scale fusion module and adds a shape flow network to correct polyp boundaries. PraNet [16] employed a reverse attention mechanism that combines region and boundary cues to emphasise polyp margin areas. The advanced feature integration component and edge‐focused attention mechanism work synergistically to rectify prediction misalignments, thus improving the segmentation precision. In addition, Tomar et al. [17] designed a dual decoder attention network based on ResUNet++ for polyp segmentation.

The FPN and its variants have been widely and successfully used in polyp segmentation tasks, as discussed above. The problems existing in FPN include (1) the successive upsampling progressively degrades fine‐grained details, weakening small target segmentation [18], and (2) the simplistic inter‐level feature fusion inadequately preserves global contextual information, hindering the model's ability to recognise complex structures [19]. To overcome these limitations, we propose a cascaded aggregation network (CAN) that systematically integrates multilevel features through progressive refinement. CAN mainly comprises three modules: the cascade aggregation module (CAM), the multiscale context‐aware module (MCAM), and the residual‐based fusion module (RFM). CAM employs a top‐down feature enhancement pathway, where high‐level features guide the progressive transmission and fusion of multiscale information, effectively strengthening the semantic representation of feature maps while maintaining spatial detail. MCAM extracts multiscale local features by employing parallel convolutional operations with varied kernel sizes and dilation rates on low‐level feature maps. By integrating conventional channel and spatial attention mechanisms, MCAM dynamically adjusts feature weights to optimise feature representation. RFM utilises a residual connection mechanism to concatenate features extracted by CAM and MCAM, enhancing the preservation of detailed features and improving segmentation precision.

In summary, the main innovations of this study include.

- We propose a CAM to enhance feature semantics via a top‐down pathway, progressively transmitting and fusing multiscale information under high‐level guidance while preserving spatial details.
- We design an MCAM for the low‐level feature extraction stage, leveraging convolutional kernels of varying sizes and dilation rates to capture multiscale local details and enhance the model's adaptability to complex backgrounds and diverse target appearances.
- We design a RFM to integrate the high‐level contextual representations extracted by CAM with the underlying features captured by MCAM to further enhance the feature expression.
- Based on CAM, MCAM and RFM, a novel end‐to‐end segmentor called CANet is proposed for the polyp segmentation task. In‐distribution and out‐of‐distribution tests show that our CANet has strong learning and generalisation capabilities.

The rest of the paper is structured as follows: Section 2 reviews related works, Section 3 presents our CANet including CAM, MCAM and RFM modules and Section 4 evaluates the performance of our CANet including both in‐distribution and out‐of‐distribution datasets. Furthermore, Section 5 provides an in‐depth discussion of the paper. Finally, Section 6 provides a summary of the work.

## 2 Related Works

### 2.1 Polyp Segmentation

Polyp segmentation technology plays a crucial role in the prevention and treatment of colorectal cancer [20]. With the rapid advancement of deep learning, its applications in medical image segmentation have expanded significantly, greatly promoting progress in polyp segmentation tasks [21]. Early polyp segmentation methods mainly relied on handcrafted feature extraction, which struggled to effectively capture global contextual information [22]. As a result, segmentation accuracy was limited when dealing with polyps of complex morphology or blurred boundaries, failing to meet the high standards required for clinical applications. In recent years, because of the continuous optimisation of deep learning models, polyp segmentation techniques have achieved remarkable breakthroughs, with significant improvements in both segmentation accuracy and robustness [23, 24]. Jha et al. [25] proposed TransNetR, an encoder‐decoder architecture that utilises a pre‐trained ResNet50 as the encoder, accompanied by three decoder modules and a final upsampling layer. This design achieves precise polyp segmentation while maintaining computational efficiency. Tomar et al. [26] integrated the self‐attention mechanism of transformers with dilated convolutions for feature fusion. This approach effectively captures local details while modelling long‐range dependencies, thereby enhancing the understanding of global semantic context in the image. Jha et al. [27] explored a combination of residual learning and hierarchical feature fusion. By incorporating residual connections, they mitigated the vanishing gradient problem in deep networks. Meanwhile, the layer‐wise fusion strategy ensured effective interaction between low‐level and high‐level features, improving the model's ability to capture both fine‐grained details and contextual information. Huang et al. [28] introduced the receptive field block (RFB) module into skip connections to enlarge the receptive fields of multi‐resolution feature maps, further boosting segmentation accuracy in polyp detection tasks.

### 2.2 Feature Pyramid Network (FPN)

Feature pyramid networks (FPN) have demonstrated outstanding performance in computer vision, particularly in the field of medical image segmentation [29]. By integrating features from multiple hierarchical levels, FPN can simultaneously capture both global semantic context and fine local details. This enhances the ability of the model to handle complex backgrounds and varying object shapes, improving segmentation accuracy and robustness [30]. In medical imaging, where precise delineation of organs and tissues is essential, FPN have emerged as a prominent research focus because of their powerful feature fusion capabilities [31]. FPN are also widely employed in polyp segmentation tasks. For instance, Dong et al. [32] proposed the polyp‐PVT model, which cascades multidepth feature maps to extend semantic information across the entire polyp region. This approach effectively fuses multiscale features while suppressing noise, thereby significantly improving segmentation performance. Zhang et al. [33] introduced a strategy that transfers local contextual characteristics from the encoder to the decoder, focussing on regions that were poorly predicted in earlier stages. Their method combines coarse‐ and fine‐grained features through a multilevel fusion scheme, resulting in more accurate boundary delineation of polyps. Furthermore, Tomar et al. [34] incorporated deep layer supervision to enhance feature learning during training. They also introduced auxiliary learning tasks to refine text embedding weights, which helped the model generalise better to polyps of varying sizes and multiple occurrences.

## 3 Methodology

### 3.1 Overall Architecture

**FIGURE 1:**
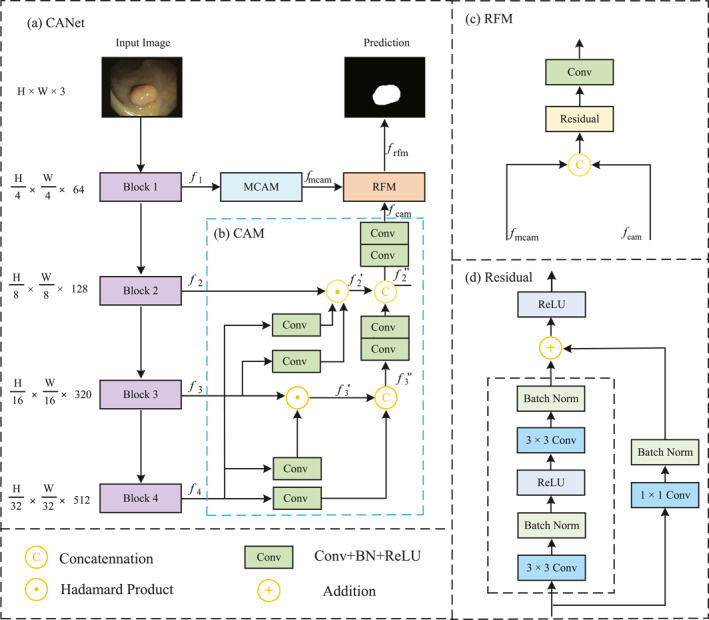
Overview of the CANet framework.

Figure 1a shows the structure of our CANet. CANet uses the backbone network to extract multiscale features ${\left\{f_{i}\right\}}_{i=1}^{4}$ from the input image $x\in R^{H\times W\times 3}$, where H and W are the high and width of the image. Here, we use pyramid vision transformer (PVT) [13] as the backbone to extract the multiscale features due to its combination of the global modelling capability of transformers and the efficient feature representation of the pyramid structure. Formally,

(1) $${\left\{f_{i}\right\}}_{i=1}^{4}=\text{PVT}\left(x\right).$$

The shallowest feature $f_{1}$ is fed into the multiscale context‐aware module (MCAM) to enhance global context:

(2) $$f_{\text{mcam}}=\text{MCAM}\left(f_{1}\right).$$

Meanwhile, the deeper features ${\left\{f_{i}\right\}}_{i=2}^{4}$ are processed by the cascaded aggregation module (CAM), which aggregates high‐level semantics in a top‐down manner.

(3) $$f_{\text{cam}}=\text{CAM}\left(f_{2},f_{3},f_{4}\right).$$

The outputs of MCAM and CAM are further fused by the residual‐based fusion module (RFM), which adaptively re‐weights and integrates the features. Formally,

(4) $$f_{\text{rfm}}=\text{RFM}\left(f_{\text{mcam}},f_{\text{cam}}\right).$$

This design allows CANet to effectively capture both global contextual dependencies and fine‐grained spatial structures, ensuring superior performance on challenging polyp segmentation tasks. The main contributions of this paper include CAM, MCAM and RFM, which are detailed in Sections 3.2, 3.3 and 3.4, respectively.

### 3.2 Cascaded Aggregation Module (CAM)

We propose CAM, a top‐down architecture that hierarchically integrates multiscale features under high‐level semantic guidance while preserving spatial details, as shown in Figure 1b. CAM comprises two aggregation stages: at each stage, high‐level semantic cues guide low‐level feature extraction, and the results are then fused with the propagated higher‐level information to produce enriched representations.

The first path takes $f_{3}$ and $f_{4}$ as inputs, using $f_{4}$ to guide feature extraction for $f_{3}$:

(5) $$f_{3}^{\prime }=Conv\left(Up\left(f_{4}\right)\right)⊙f_{3},$$

where Conv(⋅) denotes a convolution followed by batch normalisation (BN) and a non‐linear activation, whereas Up(⋅) aligns $f_{4}$’s resolution with $f_{3}$ through upsampling. Element‐wise multiplication (⊙) adaptively enhances discriminative regions in $f_{3}$ by leveraging the guidance from $f_{4}$ while suppressing less relevant features. $f_{3}^{\prime }$ is then aggregated with $f_{4}$ to obtain $f_{3}^{\prime \prime }$:

(6) $$f_{3}^{\prime \prime }=\text{Concate}\left(Conv\left(Up\left(f_{4}\right)\right),f_{3}^{\prime }\right).$$

The second path takes $f_{2}$, $f_{3}$ and $f_{4}$, as well as $f_{3}^{\prime \prime }$ (the output of the first path), as inputs and uses a similar approach to the first path to fuse these features. Unlike the first path, both $f_{3}$ and $f_{4}$ are used to guide the feature extraction of $f_{2}$, instead of relying solely on $f_{4}$. Formally,

(7) $$f_{2}^{\prime }=Conv\left({Up}^{2}\left(f_{4}\right)\right)⊙Conv\left(Up\left(f_{3}\right)\right)⊙f_{2},$$

where ${Up}^{2}\left(\cdot \right)$ denotes the upsampling operation with a scaling factor of 4. Then, similarly to Equation 6, $f_{2}^{\prime }$ is then aggregated with $f_{3}^{\prime }$ to obtain $f_{2}^{\prime \prime }$:

(8) $$f_{2}^{\prime \prime }=\text{Concate}\left(Conv\left(Conv\left(Up\left(f_{3}^{\prime }\right)\right)\right),f_{2}^{\prime }\right).$$

Ultimately, the fused feature $f_{2}^{\prime \prime }$ undergoes two convolutional layers to further transform and adjust its feature dimensions, resulting in the optimised output representation $f_{\text{cam}}$.

(9) $$f_{\text{cam}}=Conv\left(Conv\left(f_{2}^{\prime \prime }\right)\right).$$

### 3.3 Multiscale Context‐Aware Module (MCAM)

**FIGURE 2:**
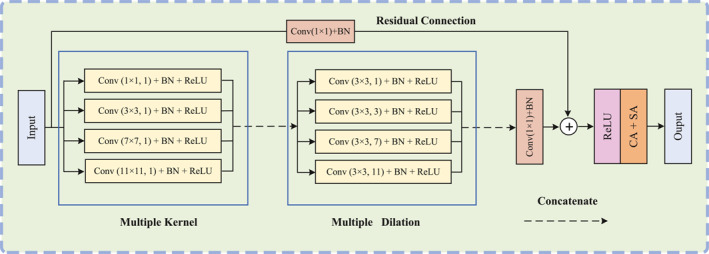
Illustration of the proposed MCAM.

We propose an MCAM to capture rich multiscale contextual information while preserving fine‐grained spatial details on the lowest‐level feature, as shown in Figure 2. The module consists of two sequentially connected branches: one using convolutions with multiple kernel sizes and the other employing dilated convolutions with varying dilation rates. Additionally, residual connections and dual attention mechanisms (channel and spatial) are incorporated to further enhance the feature representation.

In the first branch, we apply parallel convolutional operations with multiple kernel sizes to the first‐level feature $f_{1}$ (refer to Figure 1a), enabling the extraction of diverse features across different spatial scales. Formally,

(10) $$\begin{array}{cc} f_{1}^{1} & =\text{ReLU}\left(\text{BN}\left({\text{Conv}}_{1\times 1}\left(f_{1}\right)\right)\right),\\ f_{1}^{2} & =\text{ReLU}\left(\text{BN}\left({\text{Conv}}_{3\times 3}\left(f_{1}\right)\right)\right),\\ f_{1}^{3} & =\text{ReLU}\left(\text{BN}\left({\text{Conv}}_{7\times 7}\left(f_{1}\right)\right)\right),\\ f_{1}^{4} & =\text{ReLU}\left(\text{BN}\left({\text{Conv}}_{11\times 11}\left(f_{1}\right)\right)\right), \end{array}$$

where ${\text{Conv}}_{i\times i}\left(\cdot \right)$ indicates the convolution with kernel size equal to i×i. Here, convolutions with small kernels (e.g., 1×1 and 3×3) focus on local textures and fine‐grained details, whereas those with larger kernels (e.g., 7×7 and 11×11) capture broader semantic context. Batch normalisation (BN) and ReLU activation functions are applied to ensure the stability of training. Subsequently, the output features from all different kernel sizes are concatenated along the channel dimension to integrate the multiscale feature information:

(11) $$f^{\prime 1}=\text{Concate}\left(f_{1}^{1},f_{1}^{2},f_{1}^{3},f_{1}^{4}\right).$$

In the second branch, the feature map $f_{1}^{\prime }$ passes through a series of 3×3 convolutions with dilation rates d∈{1,3,7,11}, denoted as ${\text{Conv}}_{3\times 3}^{d}\left(\cdot \right)$, followed by BN and ReLU activation. Formally.

(12) $$f_{1}^{\prime 1}=\text{ReLU}\left(\text{BN}\left({\text{Conv}}_{3\times 3}^{1}\left(f_{1}^{\prime }\right)\right)\right),$$

(13) $$f_{1}^{\prime 2}=\text{ReLU}\left(\text{BN}\left({\text{Conv}}_{3\times 3}^{3}\left(f_{1}^{\prime }\right)\right)\right),$$

(14) $$f_{1}^{\prime 3}=\text{ReLU}\left(\text{BN}\left({\text{Conv}}_{3\times 3}^{7}\left(f_{1}^{\prime }\right)\right)\right),$$

(15) $$f_{1}^{\prime 4}=\text{ReLU}\left(\text{BN}\left({\text{Conv}}_{3\times 3}^{11}\left(f_{1}^{\prime }\right)\right)\right).$$

By incorporating dilated convolutions with multiple receptive fields, the model can capture contextual information at different scales while preserving spatial resolution. The resulting features are concatenated as follows:

(16) $$f_{1}^{\prime \prime }=\text{Concate}\left(f_{1}^{\prime 1},f_{1}^{\prime 2},f_{1}^{\prime 3},f_{1}^{\prime 4}\right).$$

To maintain the integrity of the original input and facilitate optimisation, we introduce a residual connection. In particular, the original feature $f_{1}$ is first transformed using a 1×1 convolution to match the dimensionality:

(17) $$f^{r}=\text{BN}\left({\text{Conv}}_{1\times 1}\left(f_{1}\right)\right).$$

$f^{r}$ is then integrated with $f_{1}^{\prime \prime }$ through summation followed by ReLU activation:

(18) $$f^{\prime }=\text{ReLU}\left(f^{r}+\text{BN}\left({\text{Conv}}_{1\times 1}\left(f_{1}^{\prime \prime }\right)\right)\right).$$

This fusion preserves low‐level spatial details from the original input while incorporating enriched contextual features. To further enhance discriminative capability, we apply sequential channel attention (CA) and spatial attention (SA) modules [35] to the fused feature $f^{\prime }$:

(19) $$f_{\text{mcam}}=\text{SA}\left(\text{CA}\left(f^{\prime }\right)\right).$$

### 3.4 Residual‐Based Fusion Module (RFM)

We propose RFM to integrate the high‐level contextual representation $f_{\text{cam}}$ extracted by CAM (refer to Section 3.2) with the underlying features captured by MCAM (refer to Section 3.3) to further enhance the feature expression. Figure 1c shows the details of the RFM. RFM first integrates $f_{\text{cam}}$ and $f_{\text{mcam}}$ through concatenation along channel dimension:

(20) $$f_{\text{con}}=\text{Concate}\left(f_{\text{cam}},f_{\text{mcam}}\right),$$

where $f_{\text{cam}}$ represents high‐level features and $f_{\text{mcam}}$ represents low‐level features. Then $f_{\text{con}}$ is further enhanced through a residual structure [36], promoting gradient flow and stabilising deep network training. In particular, $f_{\text{con}}$ is processed by two successive convolution and BN blocks, as well as Relu function between them. Formally,

(21) $$f_{x}^{\prime }=\text{BN}\left({\text{Conv}}_{3\times 3}\left(\text{ReLU}\left(\text{BN}\left({\text{Conv}}_{3\times 3}\left(f_{\text{con}}\right)\right)\right)\right)\right).$$

Meanwhile, to ensure dimension alignment for residual addition, a 1×1 convolution followed by batch normalisation is applied to the shortcut path:

(22) $$f_{\text{res}}=\text{BN}\left({\text{Conv}}_{1\times 1}\left(f_{\text{con}}\right)\right).$$

The final output of the residual block is obtained through element‐wise addition followed by a ReLU activation and convolutional module:

(23) $$f_{\text{rfm}}=\text{Conv}\left(\text{ReLU}\left(f_{x}^{\prime }+f_{\text{res}}\right)\right).$$

### 3.5 Loss Function

In the process of constructing a segmentation model, it is crucial to select the loss function appropriately. To enhance the quality and clarity of the generated saliency maps, a hybrid loss is employed for training our CANet. Formally,

(24) $$L_{\text{total}}=L_{\text{BCE}}+L_{\text{Dice}},$$

where $L_{\text{BCE}}$ denotes the binary cross‐entropy (BCE) loss function [37], which is widely used in binary classification tasks, particularly demonstrating significant advantages for imbalanced datasets. Formally,

(25) $$L_{\text{BCE}}=-\frac{1}{N}\sum_{i=1}^{N}\left[y_{i}\;\log\left(p_{i}\right)+\left(1-y_{i}\right)\log\left(1-p_{i}\right)\right].$$

$L_{\text{Dice}}$ is the Dice losss [38], which enhances model performance by computing the overlap ratio between predicted segmentation areas and ground truth annotations. Formally,

(26) $$L_{\text{Dice}}=1-\frac{2\sum_{i=1}^{N}y_{i}p_{i}}{\sum_{i=1}^{N}y_{i}+\sum_{i=1}^{N}p_{i}}.$$

## 4 Experiments

### 4.1 Experimental Setup

#### 4.1.1 Datasets

We use multiple publicly available datasets to evaluate the performance of our CANet on the polyp segmentation task, namely Kvasir‐SEG [39], PolypGen [40] and CVC‐ClinicDB [41].

Kvasir‐SEG is an open‐source gastrointestinal polyp dataset containing 1000 images with manually annotated segmentation masks, verified by experienced gastroenterologists. It supports pixel‐level segmentation tasks, particularly polyp detection in colonoscopy analysis. Image resolutions range from 332×487 to 1920×1072 and are stored in JPEG format, with the corresponding bounding boxes in the JSON files. We use 880 images for training and the remaining for testing, applying extensive data augmentation to enhance the training set.

The PolypGen dataset comprises data from over 300 patients collected across six leading medical institutions worldwide. This dataset includes single‐frame images with a resolution of 1920×1080 and video sequences at 30 fps, encompassing a total of 3762 meticulously annotated polyps. All annotations were cross‐validated by six gastrointestinal experts to ensure clinical accuracy.

CVC‐ClinicDB, released by the Computer Vision Centre at the University of Barcelona, Spain, contains 612 high‐resolution colonoscopy images (ranging from 1280×720 to 1920×1080). The dataset comprises 327 adenomatous polyps, 214 hyperplastic polyps and 71 normal mucosa samples. It features both standard and colonic distension‐enhanced image versions, along with pixel‐level annotations, lesion morphological parameters and colour histograms—providing a solid foundation for extracting features of small polyps (< 5 mm).

#### 4.1.2 Implementation Details

We implement our CANet using the PyTorch framework and conduct experiments on an NVIDIA A100 GPU system. During training, all images are resized to 256×256 pixels with a batch size of 16 over 500 epochs. The Adam optimiser is employed with an initial learning rate of 1e‐4 to regulate parameter updates. To prevent overfitting, an early stop strategy is implemented, terminating training if no performance improvement is observed on the validation set for 50 consecutive epochs. All comparative and ablation studies are performed based on the aforementioned configuration.

#### 4.1.3 Evaluation Metrics

To comprehensively evaluate the performance of CANet, we employ a variety of assessment metrics, including mean intersection over union (mIoU) [12], mean Dice similarity coefficient (mDSC) [42], recall and precision. The definitions of these metrics are as follows.

(27) $$\text{mIoU}=\frac{1}{N}\sum_{i=1}^{N}\frac{\left|A_{i}\cap B_{i}\right|}{\left|A_{i}\cup B_{i}\right|},$$

(28) $$\text{mDSC}=\frac{1}{N}\sum_{i=1}^{N}\frac{2|A_{i}\cap B_{i}|}{\left|A_{i}|+|B_{i}\right|},$$

(29) $$\text{Recall}=\frac{\mathrm{TP}}{\mathrm{TP}+\mathrm{FN}},$$

(30) $$\text{Precision}=\frac{\mathrm{TP}}{\mathrm{TP}+\mathrm{FP}}.$$

Furthermore, the incorporation of $F_{2}$‐score, a reconciled average of recall, precision and accuracy, further underscores the significance of small target detection, calculated as follows:

(31) $$F_{2}=\frac{\left(1+\beta^{2}\right)\cdot \text{precision}\cdot \text{recall}}{\beta^{2}\cdot \text{precision}+\text{recall}}.\;$$

The Hausdorff distance (HD) is also used to evaluate the precision of boundary localisation by a model, defined as follows:

(32) $$\mathrm{HD}\left(A,B\right)=\max\left(\underset{a\in A}{\sup}\underset{b\in B}{\inf}d\left(a,b\right),\underset{b\in B}{\sup}\underset{a\in A}{\inf}d\left(a,b\right)\right).$$

### 4.2 In‐Distribution Testing

**FIGURE 3:**
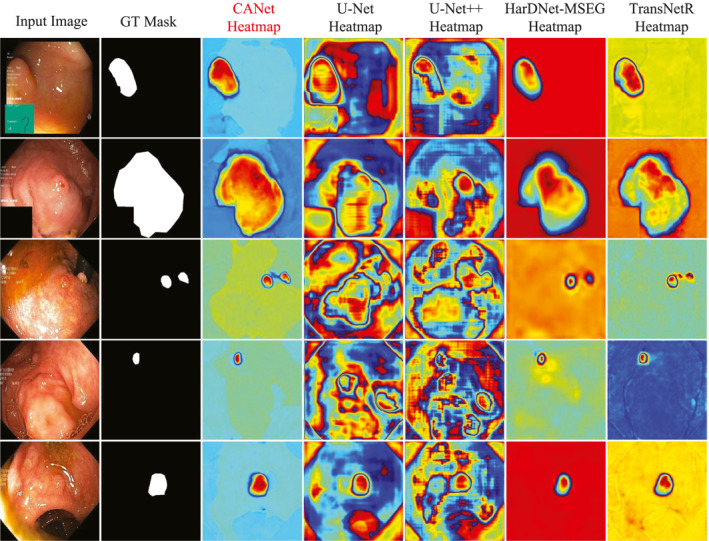
Polyp segmentation heatmap results on the Kvasir‐SEG dataset.

**FIGURE 4:**
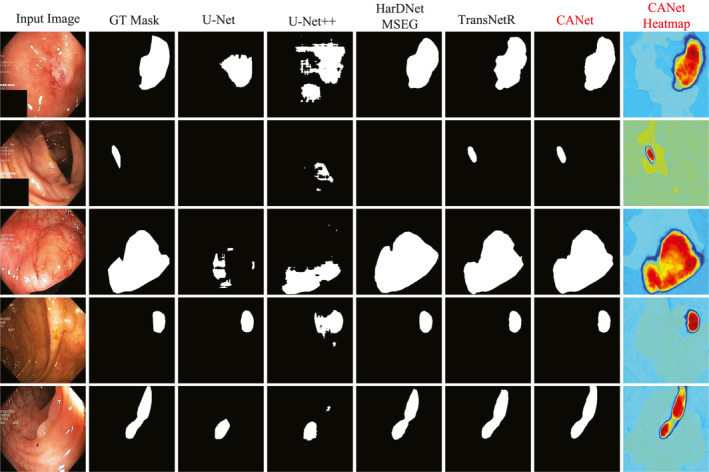
Visualisation results of polyp segmentation on the Kvasir‐SEG dataset.

**FIGURE 5:**
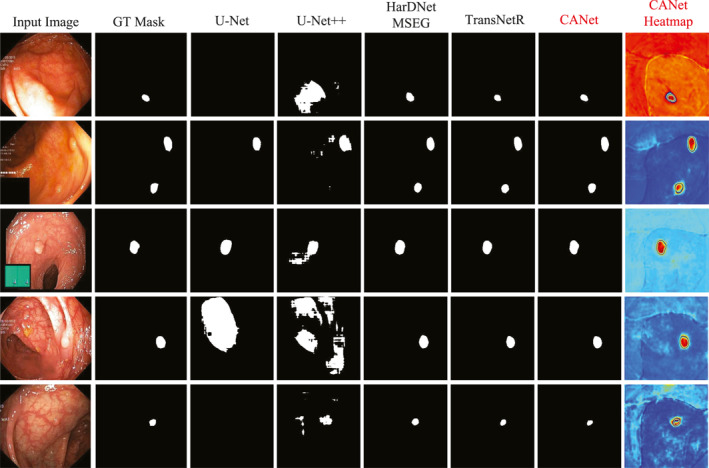
Small object polyp segmentation on Kvasir‐SEG dataset.

To evaluate the capability of the CANet model, we conducted experiments on the Kvasir‐SEG dataset. Table 1 presents the quantitative evaluation results of CANet on this dataset. Figure 3 displays the segmentation heatmaps of five methods, that is, our CANet, U‐Net, U‐Net++, HarDNet‐MSEG and TransNetR, in the Kvasir‐SEG dataset. Figures 4 and 5 further illustrate the visual segmentation results of these methods for general objects and small objects, respectively.

**TABLE 1:** Experimental results on the Kvasir‐SEG dataset.

Method	mIoU	mDSC	Recall	Prec.	F2	HD
U‐Net [5]	0.7472	0.8264	0.8504	0.8703	0.8353	4.8052
U‐Net++ [6]	0.7420	0.8228	0.8437	0.8607	0.8295	4.6904
U‐Net 3+ [7]	0.7929	0.8587	0.8518	0.9308	0.8525	4.4677
TransNetR [25]	0.8016	0.8706	0.8843	0.9073	0.8744	3.9044
TransResU‐Net [26]	0.8214	0.8884	0.9106	0.9022	0.8971	4.8971
ResU‐Net++ [43]	0.5341	0.6453	0.6964	0.7080	0.6576	4.3089
HarDNet‐MSEG [28]	0.7459	0.8260	0.8485	0.8652	0.8358	4.2036
ColonSegNet [27]	0.6980	0.7920	0.8193	0.8432	0.7999	3.9678
PVTFormer [42]	0.8153	0.9005	0.9058	0.9005	0.9037	3.4044
**Ours**	**0.8857**	**0.9361**	**0.9343**	**0.9450**	**0.9345**	**3.1666**

*Note:* The best values are highlighted in bold.

From Table 1, our CANet achieves state‐of‐the‐art performance on the Kvasir‐SEG dataset, ranking first in six key metrics: mIoU (0.8875), mDSC (0.9361), recall (0.9343), precision (0.8450), F2 (0.9345) and HD (3.1666). It is worth noting that CANet outperforms the second‐best method, PVTFormer, by a margin of 0.0704 in mIoU and 0.0356 in mDSC. In terms of recall and F2, CANet also surpasses TransResU‐Net and PVTFormer by 0.0255 and 0.0308, respectively.

As shown in Figure 3, the results in the third column indicate that the heatmaps generated by CANet not only accurately cover the entire polyp region but also clearly delineate boundary contours, demonstrating a high degree of consistency with the ground truth masks. This further validates CANet's robustness in handling polyps with complex shapes. In contrast, the heatmaps produced by U‐Net and U‐Net++ contain significant background noise and false positives; HarDNet‐MSEG sometimes fails to respond strongly to key regions; and while TransNetR shows relatively accurate localisation, its boundary precision still falls short compared to CANet.

Figure 4 shows that the results in the seventh column highlight CANet's superior performance in segmenting polyps with irregular shapes and complex structures. The segmentation outputs of U‐Net and U‐Net++ suffer from severe fragmentation and evident missed detections. Although HarDNet‐MSEG and TransNetR provide relatively complete contours, their boundary processing tends to be either overly smooth or slightly contracted. In contrast, CANet's segmentation results closely match ground truth in both shape and edge details, demonstrating excellent segmentation accuracy and boundary awareness.

In small object segmentation tasks, CANet's advantages become even more pronounced. As shown in Figure 5, the results in the seventh column clearly demonstrate its capability to detect and segment subcentimetre polyps. These small and low‐contrast targets are difficult to identify with conventional models: U‐Net and U‐Net++ fail to detect the object entirely, whereas HarDNet‐MSEG and TransNetR capture the target, but produce masks with imprecise and uneven boundaries. In contrast, CANet successfully localises the small polyp and generates a segmentation mask that aligns closely with the ground truth. The corresponding heatmap also accurately focuses on the target region, further confirming CANet's robustness and high‐resolution recognition ability in fine‐grained small object segmentation tasks.

### 4.3 Out‐of‐Distributuion Testing

**FIGURE 6:**
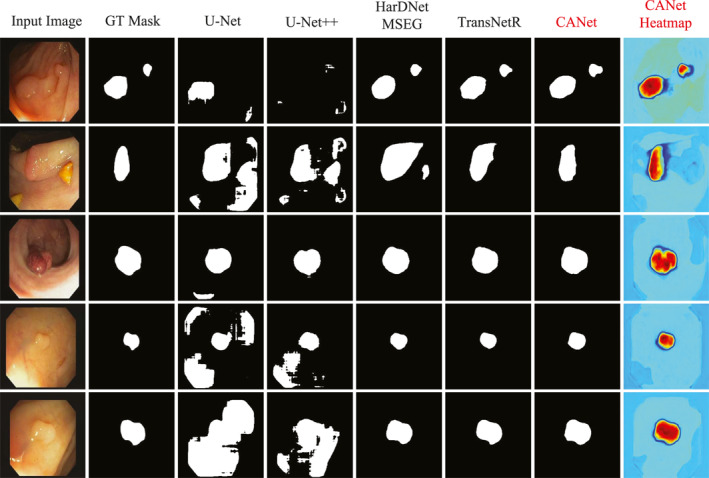
Visualisation results of polyp segmentation on the CVC‐ClinicDB dataset.

To evaluate the generalisation capability of our CANet, we perform out‐of‐distribution testing on the CVC‐ClinicDB and PolypGen datasets, using models trained on the Kvasir‐SEG dataset (Section 4.2) for comparison. Tables 2 and 3 show the quantitative evaluation results of comparing methods on the two datasets, respectively. Figure 6 displays the visual segmentation results of five methods, that is our CANet, U‐Net, U‐Net++, HarDNet‐MSEG and TransNetR, in the CVC‐ClinicDB dataset.

**TABLE 2:** Experimental results on the CVC‐ClinicDB dataset.

Method	mIoU	mDSC	Recall	Prec.	F2	HD
U‐Net [5]	0.5433	0.6336	0.6982	0.7891	0.6563	5.0396
U‐Net++ [6]	0.5475	0.6530	0.6933	0.7967	0.6556	4.9969
U‐Net 3+ [7]	0.5196	0.6039	0.7051	0.7336	0.6382	4.6382
TransNetR [25]	0.6912	0.7655	0.7571	**0.9200**	0.7565	3.9987
TransResU‐Net [26]	0.6238	0.7011	0.7794	0.7390	0.7380	4.7380
ResU‐Net++ [43]	0.3585	0.4642	0.5880	0.5770	0.5084	4.8969
HarDNet‐MSEG [28]	0.6058	0.6960	0.7173	0.8528	0.7010	4.7856
ColonSegNet [27]	0.5090	0.6126	0.6564	0.7521	0.6246	4.8697
PVTFormer [42]	0.7158	0.7889	0.7994	0.8421	0.7928	3.8719
**Ours**	**0.7225**	**0.8008**	**0.8173**	0.8389	**0.8072**	**3.6695**

*Note:* The best values are highlighted in bold.

**TABLE 3:** Experimental results on the PolypGen (C1–C6) dataset.

Method	Backbone	mIoU	mDSC	Recall	Precision	F2	HD
**C1**							
U‐Net [5]	—	0.5772	0.6469	0.6780	0.8464	0.6484	4.8983
U‐Net++ [6]	—	0.5857	0.6611	0.6953	0.8247	0.6700	4.7442
U‐Net 3+ [7]	—	0.5979	0.6631	0.6960	0.8480	0.6696	4.5920
TransNetR [25]	ResNet50	0.6538	0.7204	0.7438	**0.8778**	0.7269	4.1922
TransResU‐Net [26]	ResNet50	0.7000	0.7708	0.8137	0.8542	0.7854	4.1395
ResU‐Net++ [43]	—	0.4204	0.5239	0.6390	0.5789	0.5557	5.4847
HarDNet‐MSEG [28]	HardNet68	0.6256	0.7121	0.7800	0.7933	0.7344	4.1428
ColonSegNet [27]	—	0.5514	0.6386	0.7130	0.7423	0.6551	4.9940
PVTFormer [42]	PVT	0.7294	0.8097	**0.8985**	0.7913	0.8422	3.8923
**Ours**	PVT	**0.7747**	**0.8470**	0.8812	0.8640	**0.8557**	**3.7905**
**C2**							
U‐Net [5]	—	0.5772	0.6338	0.7347	0.7368	0.6495	4.1535
U‐Net++ [6]	—	0.5612	0.6204	0.7189	0.7631	0.6383	4.3425
U‐Net 3+ [7]	—	0.5844	0.6416	0.6970	0.7986	0.6437	4.0609
TransNetR [25]	ResNet50	0.6608	0.7203	0.8071	**0.8089**	0.7366	3.5676
TransResU‐Net [26]	ResNet50	0.7000	0.7708	0.8137	0.8542	0.7854	4.1395
ResU‐Net++ [43]	—	0.2779	0.3431	0.5003	0.4189	0.3606	5.2346
HarDNet‐MSEG [28]	HardNet68	0.5667	0.6311	0.7267	0.7149	0.6376	3.4968
ColonSegNet [27]	—	0.4659	0.5371	0.6443	0.6789	0.5439	4.5876
PVTFormer [42]	PVT	0.6467	0.7138	0.8644	0.6919	0.7502	3.4108
**Ours**	PVT	**0.7045**	**0.7647**	**0.8645**	0.8074	**0.7672**	**3.2952**
**C3**							
U‐Net [5]	—	0.6769	0.7481	0.7637	0.8787	0.7518	4.8423
U‐Net++ [6]	—	0.6530	0.7254	0.7526	0.8568	0.7332	4.7139
U‐Net 3+ [7]	—	0.6739	0.7456	0.7629	0.8703	0.7492	4.1634
TransNetR [25]	ResNet50	0.7217	0.7874	0.7904	**0.9133**	0.7863	3.7747
TransResU‐Net [26]	ResNet50	0.7516	0.8247	0.8515	0.8809	0.8346	3.7343
ResU‐Net++ [43]	—	0.4096	0.5109	0.6463	0.5484	0.5545	5.3988
HarDNet‐MSEG [28]	HardNet68	0.6623	0.7440	0.7947	0.8180	0.7619	3.6581
ColonSegNet [27]	—	0.6181	0.7064	0.7520	0.7907	0.7221	4.6104
PVTFormer [42]	PVT	0.7862	0.8596	0.9077	0.8509	0.8826	3.5109
**Ours**	PVT	**0.8105**	**0.8793**	**0.9181**	0.8945	**0.8843**	**3.4417**
**C4**							
U‐Net [5]	—	0.3699	0.4147	0.6550	0.5982	0.4263	3.1962
U‐Net++ [6]	—	0.3807	0.4202	0.6337	0.6099	0.4294	3.0279
U‐Net 3+ [7]	—	0.3909	0.4315	0.6291	0.6592	0.4386	3.0768
TransNetR [25]	ResNet50	**0.4601**	**0.5042**	0.6874	**0.7141**	**0.5096**	2.9209
TransResU‐Net [26]	ResNet50	0.4180	0.4690	0.7823	0.5472	0.4937	2.7403
ResU‐Net++ [43]	—	0.1689	0.2268	0.6342	0.2816	0.2433	3.6532
HarDNet‐MSEG [28]	HardNet68	0.3516	0.3936	0.6758	0.5535	0.4062	2.7778
ColonSegNet [27]	—	0.2933	0.3244	0.6493	0.4710	0.3558	3.1976
PVTFormer [42]	PVT	0.3585	0.4156	0.8418	0.4288	0.4502	2.5316
**Ours**	PVT	0.4328	0.5032	**0.8662**	0.5216	**0.5521**	**2.3901**
**C5**							
U‐Net [5]	—	0.2963	0.3614	0.4577	0.5497	0.3870	4.8963
U‐Net++ [6]	—	0.3143	0.3773	0.4475	0.6030	0.3935	4.6263
U‐Net 3+ [7]	—	0.3216	0.3823	0.4423	0.5927	0.3949	4.7336
TransNetR [25]	ResNet50	0.3597	0.4214	0.4508	**0.7767**	0.4232	4.4563
TransResU‐Net [26]	ResNet50	0.3352	0.4014	0.4913	0.5462	0.4213	4.7321
ResU‐Net++ [43]	—	0.2041	0.2748	0.4643	0.3027	0.3156	5.3125
HarDNet‐MSEG [28]	HardNet68	0.3090	0.3769	0.4588	0.5250	0.3970	4.4298
ColonSegNet [27]	—	0.2687	0.3416	0.4097	0.5232	0.3532	4.8444
PVTFormer [42]	PVT	0.4339	0.5125	**0.6130**	0.5461	0.5440	4.2369
**Ours**	PVT	**0.4695**	**0.5544**	0.5931	0.6489	**0.5594**	**4.1149**
**C6**							
U‐Net [5]	—	0.5384	0.6126	0.7054	0.7508	0.6362	4.3135
U‐Net++ [6]	—	0.5355	0.6163	0.7340	0.7230	0.6564	4.2843
U‐Net 3+ [7]	—	0.5387	0.6065	0.7076	0.7297	0.6351	4.0034
TransNetR [25]	ResNet50	0.6335	0.6917	0.6783	**0.9431**	0.6803	3.6173
TransResU‐Net [26]	ResNet50	0.6501	0.7151	0.7822	0.8091	0.7331	3.5544
ResU‐Net++ [43]	—	0.2816	0.3684	0.6220	0.3526	0.4326	4.9259
HarDNet‐MSEG [28]	HardNet68	0.5548	0.6341	0.7197	0.7722	0.6487	3.4179
ColonSegNet [27]	—	0.4410	0.5290	0.6199	0.6403	0.5424	4.5184
PVTFormer [42]	PVT	0.6804	0.7492	0.8413	0.7582	0.7768	3.3609
**Ours**	PVT	**0.7353**	**0.7974**	**0.8445**	0.8811	**0.8033**	**3.3393**

*Note:* The best values are highlighted in bold.

#### 4.3.1 CVC‐ClinicDB Dataset

From Table 2, our CANet achieves state‐of‐the‐art performance on the CVC‐ClinicDB dataset, ranking first in five key metrics: mIoU (0.7225), mDSC (0.8008), Recall (0.8173), F2 (0.8072), and HD (3.6695). Notably, CANet outperforms the second‐best method, PVTFormer, in mIoU and mDSC by margins of 0.0067 and 0.0119, respectively. In terms of Recall and F2, CANet outperforms both TransResU‐Net and PVTFormer by 0.0179 and 0.0144, respectively. Although CANet's Precision (0.8389) is slightly lower than that of TransNetR (0.9200), CANet delivers a well‐balanced performance across all metrics.

As shown in Figure 6, CANet (column 7) exhibits outstanding generalisation performance on out‐of‐distribution datasets. In contrast, U‐Net and U‐Net++ suffer from noticeable background noise and false positives, with several irrelevant white spots appearing in non‐polyp regions, which degrade the overall segmentation quality. HarDNet‐MSEG shows weak responses in key polyp areas, resulting in significant shrinkage of the segmentation mask and incomplete coverage of the polyp structure. Although TransNetR achieves relatively accurate localisation, its boundary processing is overly smooth, oversimplifying the actual polyp shape and leading to the loss of important structural details.

#### 4.3.2 PolypGen Dataset

As shown in Table 3, CANet demonstrates outstanding overall performance in the PolypGen dataset. Specifically, in the C1 subset, CANet achieves the highest scores in mIoU (0.7747), mDSC (0.8470), F2 (0.8557) and HD (3.7905). Similarly, on most other subsets, CANet outperforms all other models in terms of mIoU, mDSC and precision, further validating its strong generalisation capability and robustness in diverse segmentation scenarios. However, our method still has certain limitations. For example, in the highly challenging PolypGen‐C4 subset, mIoU and mDSC scores of CANet are slightly lower than those of TransNetR, indicating that there is still room for improvement when dealing with polyps that exhibit extremely low contrast or high visual similarity to the surrounding tissues.

### 4.4 Ablation Study

**FIGURE 7:**
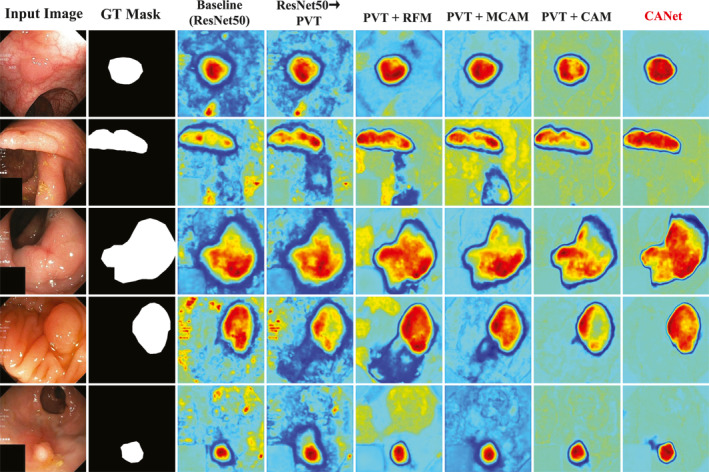
Heatmap visualisations from the ablation study on the Kvasir‐SEG dataset.

**FIGURE 8:**
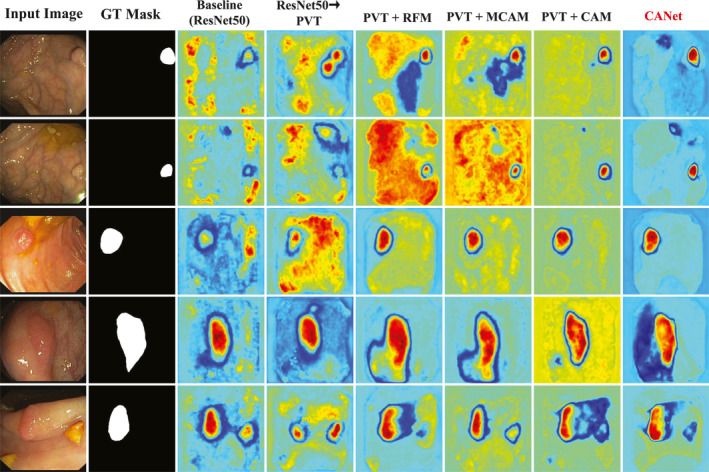
Heatmap visualisations from the ablation study on the CVC‐ClinicDB dataset.

We conducted ablation studies on the Kvasir‐SEG and CVC‐ClinicDB datasets to verify the effectiveness of each module in CANet. The corresponding quantitative results are shown in Tables 4 and 5, whereas the qualitative heatmap visualisations are presented in Figures 7 and 8, respectively. The baseline model employs ResNet‐50 as the backbone and uses upsampling and convolutional layers to generate the segmentation mask. “ResNet50→PVT” denotes the backbone ablation experiment where ResNet50 is replaced by PVT. Other experimental results correspond to module ablation studies involving RFM, MCAM and CAM.

**TABLE 4:** Ablation studies on the Kvasir‐SEG Dataset.

Method	mIoU	mDSC	Recall	Prec.	F2	HD
Baseline (ResNet50)	0.7319	0.8343	0.8584	0.8307	0.8239	4.3273
ResNet50→PVT	0.7839	0.8657	0.8869	0.8876	0.8637	4.1347
PVT + RFM	0.8536	0.8864	0.9051	0.9006	0.8999	3.9382
PVT + MCAM	0.8669	0.9042	0.9071	0.9176	0.9097	3.8987
PVT + CAM	0.8721	0.9212	0.9282	0.9399	0.9233	3.6396
**CANet**	**0.8857**	**0.9361**	**0.9343**	**0.9450**	**0.9345**	**3.1666**

*Note:* The best values are highlighted in bold.

**TABLE 5:** Ablation studies on the CVC‐ClinicDB Dataset.

Method	mIoU	mDSC	Recall	Prec.	F2	HD
Baseline (ResNet50)	0.6158	0.7089	0.7196	0.7409	0.7203	4.6125
ResNet50→PVT	0.6471	0.7329	0.7439	0.7617	0.7364	4.5194
PVT + RFM	0.6898	0.7570	0.7769	0.7869	0.7594	4.1382
PVT + MCAM	0.7069	0.7781	0.7863	0.8179	0.7747	3.8773
PVT + CAM	0.7201	0.7989	0.8064	0.8301	0.7964	3.9996
**CANet**	**0.7225**	**0.8008**	**0.8173**	**0.8389**	**0.8072**	**3.6695**

*Note:* The best values are highlighted in bold.

#### 4.4.1 Quantitative Results Analysis

From Table 4, replacing ResNet‐50 with the PVT backbone improves mIoU from 0.7319 to 0.7839 and mDSC from 0.8343 to 0.8657. With the addition of the RFM module, the mIoU and mDSC increase further to 0.8536 and 0.8864, respectively. Integrating MCAM boosts performance to 0.8669 (mIoU) and 0.9042 (mDSC), whereas the “PVT + CAM” configuration achieves 0.8721 mIoU and 0.9212 mDSC. Ultimately, the complete CANet model achieves the highest performance with an mIoU of 0.8857 and an mDSC of 0.9361.

Similarly, Table 5 shows that replacing ResNet‐50 with PVT increases mIoU from 0.6158 to 0.6471 and mDSC from 0.7089 to 0.7329. With RFM, these metrics improve to 0.6898 and 0.7570, respectively. Adding MCAM further raises them to 0.7069 and 0.7781, and the “PVT + CAM” combination achieves 0.7201 mIoU and 0.7989 mDSC. The full CANet model achieves the best results with an mIoU of 0.7225 and an mDSC of 0.8008.

#### 4.4.2 Qualitative Heatmap Analysis

Figure 7 illustrates that replacing ResNet50 with PVT in the backbone ablation significantly enhances the model's response in polyp regions, resulting in more continuous boundary responses and more precise attention to polyps. In module ablation experiments, PVT combined with RFM improves multiscale texture detail capture while maintaining global perception, especially enhancing edge responses. PVT with MCAM concentrates on key structural areas with clear contours but has limitations in fine. PVT with CAM excels in global context modelling, offering broader coverage but less detailed depiction. When all three modules (RFM, MCAM and CAM) are integrated as CANet, the heatmap balances global and local features with clear contours and smooth edge transitions, reflecting their complementary advantages.

Figure 8 shows that, in backbone network ablation experiments, models using ResNet50 or PVT exhibit weak responses to polyp regions and produce a large number of misclassifications. In module ablation experiments, integrating RFM yields modest improvements in boundary delineation, though background interference remains noticeable. Incorporating MCAM strengthens the depiction of local details, yet the overall structure remains ambiguous. Adding CAM results in more continuous and complete contours, but fine structural elements are still not fully preserved. When RFM, MCAM and CAM are integrated into the complete CANet, the generated heatmaps display sharp and well‐aligned boundaries that closely match the ground truth, illustrating that the combined effect of these modules markedly boosts segmentation precision.

## 5 Discussion

Colorectal cancer (CRC) remains one of the leading causes of cancer‐related mortality worldwide, making early and accurate detection of polyps critical for improving patient outcomes. Although traditional feature pyramid network (FPN) based architectures have contributed to advances in polyp segmentation, their limited capacity to preserve fine‐grained details and capture global contextual information constrains their performance, particularly when segmenting small or morphologically complex polyps.

The proposed CANet addresses these limitations by leveraging the complementary strengths of three key modules: cascaded aggregation module (CAM), multiscale context‐aware module (MCAM), and residual‐based fusion module (RFM). CAM strengthens the representation of small polyps by performing multiscale feature aggregation guided by high‐level semantics from the backbone. This facilitates the suppression of background noise while enhancing target localisation and structural clarity, particularly around boundaries and internal textures. The MCAM expands the receptive field through the use of dilated convolutions and a multibranch structure, enabling the extraction of contextual features at multiple scales without compromising spatial resolution. This is crucial for capturing subtle morphological variations in small polyps. The RFM further bridges the semantic gap between high‐level and low‐level features by fusing CAM‐derived semantic context with MCAM captured local detail. This residual‐based fusion ensures effective information preservation and feature complementarity. Experimental results in both in‐distribution (Kvasir‐SEG) and out‐of‐distribution datasets (CVC‐ClinicDB and PolypGen) confirm CANet's superior segmentation performance. In particular, CANet consistently outperforms strong baseline models, including U‐Net, U‐Net++, PVTFormer and TransNetR in key evaluation metrics.

**FIGURE 9:**
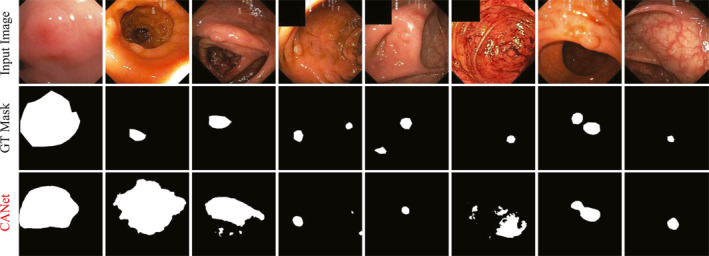
Failure cases of CANet on the Kvasir‐SEG dataset.

Extensive experiments demonstrate that CANet achieves excellent performance in‐distribution and out‐of‐distribution tests; however, it still struggles with polyps that exhibit very low contrast against the surrounding tissues. As shown in Figure 9, when the texture and colour of a polyp are very similar to those of adjacent tissue regions, our CANet exhibits insufficient feature discrimination, causing missed detections or inaccurate boundaries, especially for small polyps. The possible reason is that CANet employs a transformer backbone that uses self‐attention to dynamically capture global dependencies but has lower sensitivity to subtle texture differences and local details, hindering separation from similar backgrounds, and the which may lead to overlooking local structural information, especially for small polyps. Moreover, the loss of such detailed information may be progressively amplified during the process of feature fusion, which, in turn, further weakens the representation of small polyps and adversely affects segmentation accuracy.

Future work will focus on enhancing both feature extraction and feature fusion to address the limitations of CANet in very low contrast polyp segmentation. In terms of feature extraction, future efforts will explore the integration of local attention mechanisms or lightweight convolutional modules to improve the perception of subtle texture details and small objects. High‐frequency information, such as edges and fine structural patterns, will also be leveraged to better delineate polyp contours and mitigate confusion with the background. Regarding feature fusion, we will investigate multiscale fusion strategies combined with cross‐scale attention and adaptive pooling to better integrate global semantic context with local details, as well as boundary‐aware fusion mechanisms to preserve shape and edge information.

## 6 Conclusion

This paper proposes a novel cascaded aggregation network (CANet) to improve the precision and robustness of colorectal polyp segmentation. CANet employs the PVT Transformer as the backbone and integrates a cascaded aggregation Module (CAM), a multiscale context‐aware module (MCAM), and a residual‐based fusion module (RFM) to address the limitations of traditional feature pyramid networks in detecting small targets and capturing global contextual information. By introducing a top‐down semantic enhancement pathway, CAM enhances semantic feature representation while preserving spatial detail. MCAM utilises convolutional operations with varied kernel sizes and dilation rates to improve the model's ability to perceive local details in complex scenarios, whereas the RFM leverages residual mechanisms to optimise multi‐level feature fusion, preserving edge details and improving overall segmentation performance. Experimental results on multiple public datasets demonstrate that CANet consistently outperforms existing state‐of‐the‐art methods across various evaluation metrics, particularly excelling in scenarios involving small targets and blurred boundaries.

## Author Contributions

**Yanru Jia**: writing – original draught, review and editing. **Yu Zeng**: software, writing – review and editing. **Huaping Guo**: methodology, writing – review and editing.

## Conflicts of Interest

The authors declare no conflicts of interest.

## Data Availability

The source code is available at https://github.com/hpguo1982/CANet.
